# 3,6-Dibromo­phenanthrene

**DOI:** 10.1107/S1600536812041621

**Published:** 2012-10-10

**Authors:** Ruri Yokota, Chitoshi Kitamura, Takeshi Kawase

**Affiliations:** a Department of Materials Science and Chemistry, Graduate School of Engineering, University of Hyogo, 2167 Shosha, Himeji, Hyogo 671-2280, Japan

## Abstract

The phenanthrene ring in the title compound, C_14_H_8_Br_2_, is approximately planar [maximum deviation = 0.039 (3) Å]. In contrast, the two bromo atoms are displaced slightly from the phenanthrene plane [maximum deviation = 0.1637 (3) Å]. In the crystal, the mol­ecules adopt a herringbone-like arrangement and form face-to-face slipped π–π stacking inter­actions along the *b* axis, with an inter­planar distance of 3.544 (3) Å and slippage of 1.81 Å. The crystal studied was a racemic twin with a minor twin fraction of 0.390 (10).

## Related literature

For the synthesis of the title compound using the improved photocyclization of 4,4′-dibromo-*trans*-stilbene, see: Talele *et al.* (2009 ▶). For the original synthesis and applications of the title compound, see: Nakamura *et al.* (1996 ▶).
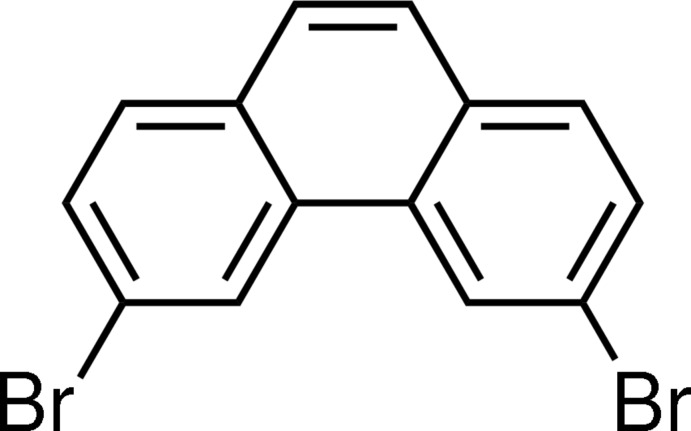


## Experimental

### 

#### Crystal data

C_14_H_8_Br_2_
*M* *_r_* = 336.02
Monoclinic, 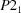

*a* = 6.8697 (5) Å
*b* = 3.9809 (2) Å
*c* = 20.5002 (11) Å
β = 93.813 (2)°
*V* = 559.39 (6) Å^3^
*Z* = 2
Mo *K*α radiation
μ = 7.21 mm^−1^
*T* = 223 K
0.62 × 0.08 × 0.03 mm

#### Data collection

Rigaku R-AXIS RAPID diffractometer
Absorption correction: numerical (*NUMABS*; Higashi, 1999 ▶) *T*_min_ = 0.196, *T*_max_ = 0.793
5372 measured reflections
2267 independent reflections
2084 reflections with *I* > 2σ(*I*)
*R*_int_ = 0.022

#### Refinement

*R*[*F* ^2^ > 2σ(*F* ^2^)] = 0.018
*wR*(*F* ^2^) = 0.037
*S* = 1.00
2267 reflections
146 parameters
1 restraint
H-atom parameters constrained
Δρ_max_ = 0.38 e Å^−3^
Δρ_min_ = −0.46 e Å^−3^
Absolute structure: Flack (1983 ▶), 831 Friedel pairs
Flack parameter: 0.390 (10)

### 

Data collection: *RAPID-AUTO* (Rigaku, 1999 ▶); cell refinement: *PROCESS-AUTO* (Rigaku, 1998 ▶); data reduction: *PROCESS-AUTO*; program(s) used to solve structure: *SIR2004* (Burla *et al.*, 2005 ▶); program(s) used to refine structure: *SHELXL97* (Sheldrick, 2008 ▶); molecular graphics: *ORTEP-3 for Windows* (Farrugia, 1997 ▶); software used to prepare material for publication: *WinGX* (Farrugia, 1999 ▶).

## Supplementary Material

Crystal structure: contains datablock(s) global, I. DOI: 10.1107/S1600536812041621/qk2043sup1.cif

Structure factors: contains datablock(s) I. DOI: 10.1107/S1600536812041621/qk2043Isup2.hkl

Supplementary material file. DOI: 10.1107/S1600536812041621/qk2043Isup3.cml

Additional supplementary materials:  crystallographic information; 3D view; checkCIF report

## Figures and Tables

**Fig. 1.**
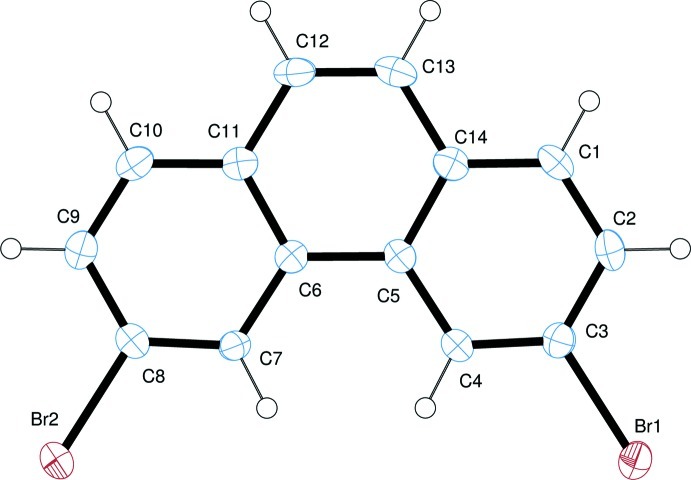
The molecular structure of (I), showing the atomic numbering and 40% probability displacement ellipsoids.

**Fig. 2.**
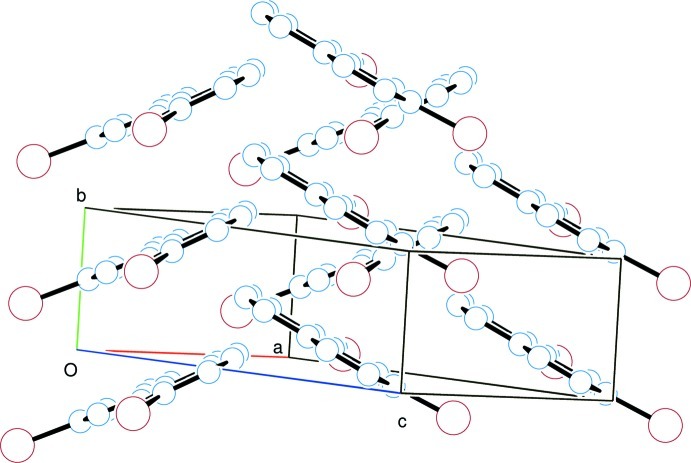
The packing diagram of (I). Hydrogen atoms are omitted for clarity.

## supplementary crystallographic information

### Comment

Phenanthrene is a polycylic aromatic hydrocarbon (PAH) as well as a potential building block for higher-order π-extended PAHs. The title compound, 3,6-dibromophenanthrene, was first prepared by Nakamura *et al.* (1996). The bromo functional group on the aromatic ring is a suitable substrate for a variety of cross-coupling reaction. Recently, the improved synthesis was reported by Talele *et al.* (2009). However, the X-ray structure was not reported to date. We report herein the crystal structure of the title compound, (I).

The molecular structure of (I) is shown in Fig. 1. The crystal was a racemic twin with a minor twin fraction of 0.390 (10). The molecule is approximately planar except for Br1 and Br2 [the maximum deviation is 0.1637 (3) Å for Br2]. The bonds lengths and angles are in good agreement with the standard values. As shown in Fig. 2, the crystal structure is characterized by a combination of a columnar stacking and a herrinbone-like arrangement. Along the *b* axis, there are two columns per unit cell in which the molecules form face-to-face slipped π-stacks with an interplanar distance of 3.543 Å. The interplanar tilt angle between the phenanthrene rings in two adjacent columns is 54.21°.

### Experimental

The title compound was prepared from 4,4'-dibromo-*trans*-stilbene according to the literature procedure of Talele *et al.* (2009). The title compound was dissolved in hot hexane. After cooling of the solution to room temperature, single crystals suitable for X-ray analysis were obtained.

### Refinement

All the aromatic H atoms were positioned geometrically and refined using a riding model with C—H = 0.94 Å and *U*_iso_(H) = 1.2*U*_eq_(C). In final refinement cycles, racemic twinning was taken into account with a TWIN and a BASF instruction of program *SHELXL97* (Sheldrick, 2008), giving a minor twin fraction of 0.390 (10).

### Crystal data

**Table:**

C_14_H_8_Br_2_	*F*(000) = 324
*M**_r_* = 336.02	*D*_x_ = 1.995 Mg m^−^^3^
Monoclinic, *P*2_1_	Mo *K*α radiation, λ = 0.71073 Å
Hall symbol: P 2yb	Cell parameters from 4191 reflections
*a* = 6.8697 (5) Å	θ = 3.1–27.5°
*b* = 3.9809 (2) Å	µ = 7.21 mm^−^^1^
*c* = 20.5002 (11) Å	*T* = 223 K
β = 93.813 (2)°	Needle, colorless
*V* = 559.39 (6) Å^3^	0.62 × 0.08 × 0.03 mm
*Z* = 2	

### Data collection

**Table:**

Rigaku R-AXIS RAPID diffractometer	2267 independent reflections
Radiation source: fine-focus sealed x-ray tube	2084 reflections with *I* > 2σ(*I*)
Graphite monochromator	*R*_int_ = 0.022
Detector resolution: 10 pixels mm^-1^	θ_max_ = 27.5°, θ_min_ = 3.1°
ω scans	*h* = −8→8
Absorption correction: numerical (*NUMABS*; Higashi, 1999)	*k* = −5→4
*T*_min_ = 0.196, *T*_max_ = 0.793	*l* = −26→26
5372 measured reflections	

### Refinement

**Table:**

Refinement on *F*^2^	Secondary atom site location: difference Fourier map
Least-squares matrix: full	Hydrogen site location: inferred from neighbouring sites
*R*[*F*^2^ > 2σ(*F*^2^)] = 0.018	H-atom parameters constrained
*wR*(*F*^2^) = 0.037	*w* = 1/[σ^2^(*F*_o_^2^) + (0.0175*P*)^2^] where *P* = (*F*_o_^2^ + 2*F*_c_^2^)/3
*S* = 1.00	(Δ/σ)_max_ = 0.002
2267 reflections	Δρ_max_ = 0.38 e Å^−^^3^
146 parameters	Δρ_min_ = −0.46 e Å^−^^3^
1 restraint	Absolute structure: Flack (1983), 831 Friedel pairs
Primary atom site location: structure-invariant direct methods	Flack parameter: 0.390 (10)

### Special details

**Table:**

Geometry. All e.s.d.'s (except the e.s.d. in the dihedral angle between two l.s. planes) are estimated using the full covariance matrix. The cell e.s.d.'s are taken into account individually in the estimation of e.s.d.'s in distances, angles and torsion angles; correlations between e.s.d.'s in cell parameters are only used when they are defined by crystal symmetry. An approximate (isotropic) treatment of cell e.s.d.'s is used for estimating e.s.d.'s involving l.s. planes.
Refinement. Refinement of *F*^2^ against ALL reflections. The weighted *R*-factor *wR* and goodness of fit *S* are based on *F*^2^, conventional *R*-factors *R* are based on *F*, with *F* set to zero for negative *F*^2^. The threshold expression of *F*^2^ > σ(*F*^2^) is used only for calculating *R*-factors(gt) *etc*. and is not relevant to the choice of reflections for refinement. *R*-factors based on *F*^2^ are statistically about twice as large as those based on *F*, and *R*- factors based on ALL data will be even larger.

### Fractional atomic coordinates and isotropic or equivalent isotropic displacement parameters (Å^2^)

**Table:**

	*x*	*y*	*z*	*U*_iso_*/*U*_eq_	
C1	−0.1808 (4)	0.2590 (6)	0.88179 (11)	0.0315 (7)	
H1	−0.2947	0.3158	0.9023	0.038*	
C2	−0.0292 (4)	0.1043 (7)	0.91762 (12)	0.0313 (6)	
H2	−0.0389	0.0537	0.9621	0.038*	
C3	0.1384 (4)	0.0249 (7)	0.88644 (11)	0.0270 (6)	
C4	0.1569 (4)	0.0897 (6)	0.82192 (11)	0.0249 (6)	
H4	0.2724	0.0318	0.8025	0.03*	
C5	0.0013 (4)	0.2449 (6)	0.78399 (11)	0.0243 (6)	
C6	0.0120 (3)	0.3192 (8)	0.71506 (10)	0.0236 (5)	
C7	0.1731 (4)	0.2289 (6)	0.67943 (11)	0.0241 (6)	
H7	0.2806	0.1184	0.7005	0.029*	
C8	0.1728 (4)	0.3020 (7)	0.61427 (11)	0.0259 (5)	
C9	0.0176 (4)	0.4707 (7)	0.58081 (12)	0.0301 (6)	
H9	0.0215	0.522	0.5362	0.036*	
C10	−0.1392 (4)	0.5590 (7)	0.61443 (12)	0.0309 (6)	
H10	−0.2438	0.6729	0.5924	0.037*	
C11	−0.1487 (4)	0.4842 (7)	0.68117 (11)	0.0260 (5)	
C12	−0.3169 (4)	0.5686 (7)	0.71513 (13)	0.0325 (7)	
H12	−0.4223	0.6774	0.6925	0.039*	
C13	−0.3277 (4)	0.4956 (8)	0.77907 (13)	0.0330 (6)	
H13	−0.4408	0.5519	0.8001	0.04*	
C14	−0.1683 (4)	0.3330 (8)	0.81549 (11)	0.0270 (5)	
Br1	0.35013 (4)	−0.17379 (7)	0.937295 (11)	0.03280 (8)	
Br2	0.38478 (4)	0.16231 (7)	0.565973 (12)	0.03364 (8)	

### Atomic displacement parameters (Å^2^)

**Table:**

	*U*^11^	*U*^22^	*U*^33^	*U*^12^	*U*^13^	*U*^23^
C1	0.0312 (15)	0.0336 (18)	0.0310 (12)	0.0016 (11)	0.0128 (11)	−0.0049 (11)
C2	0.0392 (16)	0.0317 (16)	0.0237 (11)	−0.0038 (12)	0.0084 (11)	0.0023 (12)
C3	0.0291 (15)	0.0259 (12)	0.0256 (12)	−0.0017 (11)	0.0000 (11)	−0.0018 (11)
C4	0.0248 (14)	0.0252 (15)	0.0251 (11)	−0.0008 (10)	0.0054 (10)	−0.0017 (10)
C5	0.0248 (13)	0.0239 (15)	0.0245 (11)	−0.0031 (9)	0.0029 (10)	−0.0029 (10)
C6	0.0229 (12)	0.0214 (10)	0.0262 (11)	−0.0041 (13)	0.0002 (9)	−0.0010 (13)
C7	0.0222 (12)	0.0258 (15)	0.0241 (11)	0.0026 (9)	−0.0001 (9)	0.0001 (10)
C8	0.0265 (13)	0.0248 (12)	0.0268 (11)	−0.0043 (12)	0.0047 (10)	−0.0039 (12)
C9	0.0363 (16)	0.0281 (13)	0.0255 (12)	−0.0004 (13)	−0.0007 (11)	0.0012 (12)
C10	0.0296 (16)	0.0299 (14)	0.0324 (13)	0.0032 (11)	−0.0053 (11)	0.0001 (12)
C11	0.0247 (14)	0.0228 (12)	0.0301 (12)	−0.0018 (11)	−0.0009 (10)	−0.0032 (12)
C12	0.0223 (15)	0.0348 (16)	0.0396 (14)	0.0064 (11)	−0.0028 (12)	−0.0063 (13)
C13	0.0237 (15)	0.0344 (14)	0.0416 (14)	0.0030 (13)	0.0071 (11)	−0.0080 (14)
C14	0.0255 (13)	0.0244 (11)	0.0315 (11)	−0.0029 (14)	0.0040 (10)	−0.0012 (14)
Br1	0.03496 (16)	0.03660 (14)	0.02647 (12)	0.00097 (14)	−0.00083 (10)	0.00312 (13)
Br2	0.03388 (15)	0.04005 (15)	0.02791 (12)	0.00275 (14)	0.00893 (10)	0.00034 (13)

### Geometric parameters (Å, º)

**Table:**

C1—C2	1.379 (4)	C7—C8	1.367 (3)
C1—C14	1.399 (3)	C7—H7	0.94
C1—H1	0.94	C8—C9	1.400 (3)
C2—C3	1.390 (4)	C8—Br2	1.898 (2)
C2—H2	0.94	C9—C10	1.363 (4)
C3—C4	1.362 (3)	C9—H9	0.94
C3—Br1	1.904 (2)	C10—C11	1.406 (3)
C4—C5	1.420 (3)	C10—H10	0.94
C4—H4	0.94	C11—C12	1.428 (4)
C5—C14	1.413 (3)	C12—C13	1.350 (4)
C5—C6	1.450 (3)	C12—H12	0.94
C6—C7	1.412 (3)	C13—C14	1.437 (4)
C6—C11	1.426 (3)	C13—H13	0.94
			
C2—C1—C14	121.2 (2)	C7—C8—C9	122.2 (2)
C2—C1—H1	119.4	C7—C8—Br2	119.72 (19)
C14—C1—H1	119.4	C9—C8—Br2	118.07 (17)
C1—C2—C3	118.4 (2)	C10—C9—C8	118.5 (2)
C1—C2—H2	120.8	C10—C9—H9	120.7
C3—C2—H2	120.8	C8—C9—H9	120.7
C4—C3—C2	122.5 (2)	C9—C10—C11	121.8 (2)
C4—C3—Br1	119.6 (2)	C9—C10—H10	119.1
C2—C3—Br1	117.94 (17)	C11—C10—H10	119.1
C3—C4—C5	119.9 (2)	C6—C11—C10	119.1 (2)
C3—C4—H4	120	C6—C11—C12	119.8 (2)
C5—C4—H4	120	C10—C11—C12	121.1 (2)
C14—C5—C4	118.1 (2)	C13—C12—C11	121.4 (2)
C14—C5—C6	119.4 (2)	C13—C12—H12	119.3
C4—C5—C6	122.5 (2)	C11—C12—H12	119.3
C7—C6—C11	118.3 (2)	C12—C13—C14	120.8 (3)
C7—C6—C5	123.0 (2)	C12—C13—H13	119.6
C11—C6—C5	118.7 (2)	C14—C13—H13	119.6
C8—C7—C6	120.0 (2)	C5—C14—C1	119.8 (2)
C8—C7—H7	120	C5—C14—C13	119.8 (2)
C6—C7—H7	120	C1—C14—C13	120.4 (2)
			
C14—C1—C2—C3	0.5 (4)	C7—C6—C11—C10	−1.7 (4)
C1—C2—C3—C4	−1.0 (4)	C5—C6—C11—C10	179.6 (2)
C1—C2—C3—Br1	177.77 (19)	C7—C6—C11—C12	177.8 (2)
C2—C3—C4—C5	0.2 (4)	C5—C6—C11—C12	−0.8 (4)
Br1—C3—C4—C5	−178.56 (17)	C9—C10—C11—C6	1.6 (4)
C3—C4—C5—C14	1.1 (3)	C9—C10—C11—C12	−177.9 (3)
C3—C4—C5—C6	−179.8 (2)	C6—C11—C12—C13	0.0 (4)
C14—C5—C6—C7	−177.7 (3)	C10—C11—C12—C13	179.5 (3)
C4—C5—C6—C7	3.2 (4)	C11—C12—C13—C14	0.7 (5)
C14—C5—C6—C11	0.9 (4)	C4—C5—C14—C1	−1.6 (4)
C4—C5—C6—C11	−178.2 (2)	C6—C5—C14—C1	179.3 (3)
C11—C6—C7—C8	0.5 (4)	C4—C5—C14—C13	179.0 (2)
C5—C6—C7—C8	179.0 (2)	C6—C5—C14—C13	−0.2 (4)
C6—C7—C8—C9	1.0 (4)	C2—C1—C14—C5	0.8 (4)
C6—C7—C8—Br2	−177.3 (2)	C2—C1—C14—C13	−179.7 (3)
C7—C8—C9—C10	−1.1 (4)	C12—C13—C14—C5	−0.6 (5)
Br2—C8—C9—C10	177.1 (2)	C12—C13—C14—C1	179.9 (3)
C8—C9—C10—C11	−0.2 (4)		
